# Idebenone improves motor dysfunction, learning and memory by regulating mitophagy in MPTP-treated mice

**DOI:** 10.1038/s41420-022-00826-8

**Published:** 2022-01-17

**Authors:** Junqiang Yan, Wenjie Sun, Mengmeng Shen, Yongjiang Zhang, Menghan Jiang, Anran Liu, Hongxia Ma, Xiaoyi Lai, Jiannan Wu

**Affiliations:** 1 Neuromolecular Biology Laboratory, The First Affiliated Hospital, and College of Clinical Medicine of Henan University of Science and Technology, Luoyang, 471003 China; 2 Department of Neurology, The First Affiliated Hospital, and College of Clinical Medicine of Henan University of Science and Technology, Luoyang, 471003 China

**Keywords:** Parkinson's disease, Cell death in the nervous system, Target validation

## Abstract

The progression of Parkinson’s disease (PD) is often accompanied by the loss of substantia nigra dopaminergic neurons, mitophagy damage, learning, and memory impairment. Idebenone is a therapeutic drug that targets the mitochondria of neurodegenerative diseases, but its role in Parkinson’s disease and its pathological mechanism are still unclear. The purpose of this study was to investigate whether idebenone could improve behavioral disorders, especially motor, learning, and memory disorders, in mouse PD models and to explore its molecular mechanism. In the present study, C57BL-6 mice underwent intraperitoneal injection of MPTP (30 mg/kg) once a day for five consecutive days. Then, a 200 mg/kg dose was given as a single daily gavage of idebenone dissolved in water for 21 days after the successful establishment of the subacute MPTP model. Motor, learning, and memory were measured by a water maze and a rotarod test. Our results showed that idebenone could reduce MPTP-induced dopaminergic neuron damage and improve movement disorders, memory, and learning ability, which may be associated with upregulating mitochondrial autophagy-related outer membrane proteins VDAC1 and BNIP3 and activating the Parkin/PINK1 mitochondrial autophagy pathway. To confirm whether idebenone promotes the smooth progression of autophagy, we used eGFP-mCherry-LC3 mice to construct a subacute model of Parkinson’s disease and found that idebenone can increase autophagy in dopaminergic neurons in Parkinson’s disease. In summary, our results confirm that idebenone can regulate the expression of the mitochondrial outer membrane proteins VDAC1 and BNIP3, activate Parkin/PINK1 mitophagy, promote the degradation of damaged mitochondria, reduce dopaminergic neuron damage, and improve behavioral disorders in Parkinson’s disease mice.

## Introduction

Parkinson’s disease (PD) is a progressive neurodegenerative disease characterized by mitochondrial dysfunction, proteasome damage, and apoptotic α-synuclein aggregation [1]. The clinical manifestations mainly include resting tremor, bradykinesia, muscle rigidity, and postural and gait disorders, which seriously affect the quality of life of the patients. Mitochondrial dysfunction of dopaminergic neurons is the main pathological mechanism in Parkinson’s disease [2, 3]. Mitophagy disorder is the main factor in mitochondrial dysfunction, affecting the quality of the mitochondria [4] and the survival efficiency of cells in PD [5]. Some studies have shown that dopaminergic drugs can improve learning and memory [6]. However, drugs that improve mitochondrial function in Parkinson’s disease are rare.

Parkin and PINK1 are related to autophagy, and autophagy of damaged mitochondria in mammals is mainly mediated by the PINK1/Parkin pathway [7]. PINK1 is a mitochondrial outer membrane protein, and Parkin is an E3 ubiquitin ligase. Mutations in PINK1 and Parkin are related to inherited PD. In PD cell models, such as MPP^+^ oxidative stress treatment of mitochondrial damage, PINK1 is recruited to the damaged mitochondrial outer membrane, and the PINK1/Parkin pathway is triggered. It can promote the recruitment of Parkin in the cytoplasm to damaged mitochondria and catalyze the ubiquitination of mitochondrial outer membrane proteins. Ubiquitin junction protein P62 is subsequently recruited to the mitochondria through its C-terminal ubiquitin-binding domain and it binds to the ubiquitin on proteins. Subsequently, it is recruited to the new autophagosome and degraded by its N-terminal binding to the marker protein LC3 on the autophagy membrane [8]. Increased LC3-II/LC3-I suggests enhanced autophagy, which can degrade damaged mitochondria more efficiently [9]. BNIP3 is a mitochondrial outer membrane protein that regulates mitochondrial autophagy by binding to the autophagy protein LC3 [10]. VDAC1 participates in cell volume regulation and apoptosis in the plasma membrane through mitochondrial outer membrane and plasma membrane formation channels. In depolarized mitochondria, VDAC1 promotes mitophagy or inhibits apoptosis by acting downstream of Parkin and PINK1 [11].

Idebenone is a drug that can improve brain energy metabolism and it has a mild hypotensive effect. It is usually used for cerebral infarction, cerebral hemorrhage, and atherosclerosis sequelae caused by brain dysfunction, low awareness, emotional disorders, language disorders, dementia, and other conditions. It also has a strong antioxidant effect [12]. Idebenone can provide electrons to reduce the effects of free radicals, as well as to support the mitochondrial respiratory chains, to assist in ATP production. There is evidence that idebenone can affect the expression of mitochondrial complexes, thereby offsetting mitochondrial dysfunction to some extent [13]. However, these effects alone cannot easily explain the normalization of idebenone-dependent mitochondrial function under various pathological conditions.

Related studies have shown that idebenone can improve neurological deficits in PD by inhibiting neuroinflammation and regulating the microglial phenotype [14]. However, high concentrations of idebenone in SH-SY5Y cells can induce BAX to increase caspase-3, leading to apoptosis. At the same time, idebenone has a protective effect on rotenone injury and has multiple effects on cell oxidase and lipid peroxidation [15].

Therefore, we used MPTP to establish PD subacute model mice and treated them with idebenone to study their changes in behavior and mitochondrial autophagy function. Idebenone significantly improved the level of mitophagy, improved the motor dysfunction of the mice, and improved their conditional learning and memory ability.

## Results

### Idebenone improves MPTP-induced dopaminergic neuronal damage in the substantia nigra

**Fig. 1:**
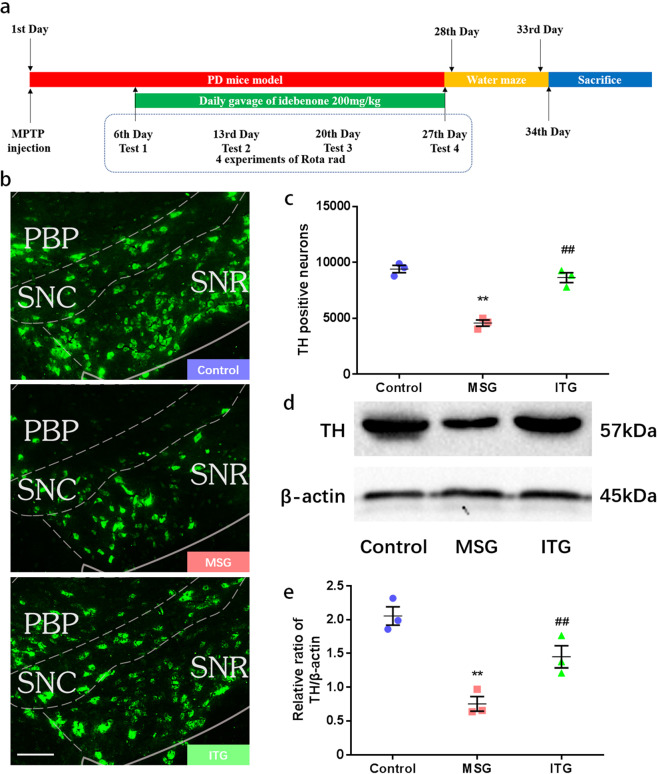
Effect of idebenone on dopaminergic neurons in the substantia nigra of an MPTP-induced subacute PD mouse model. **a** PD mouse model, idebenone treatment and behavioral processing diagram. **b** Immunofluorescence of dopaminergic neurons in the substantia nigra of the mice. Bar = 200 μm *N* = 3 **c** Statistics of dopaminergic neurons in the substantia nigra of the mice. **d** Protein immunoblotting of TH levels in the substantia nigra of the mice. **e** Statistical analysis of TH protein levels in the substantia nigra of the mice. **P < 0.01 compared with the control, ^##^*P* < 0.01 compared with MSG, *N* = 3.

MPTP is a commonly used reagent for the establishment of the PD mouse model. MPTP dehydrogenation in substantia nigra cells generates MPP+, affecting their mitochondrial function and leading to the loss of dopaminergic neurons in the substantia nigra, which is one of the most typical pathological manifestations of PD. TH is a key enzyme in dopamine formation that can convert l-tyrosine into L-3,4-dihydroxyphenylalanine (L-DOPA) by tyrosine 3-monooxygenase (tyrosine 3-monooxygenase). We established an MPTP-induced subacute PD animal model as shown in Fig. 1a. After MPTP induction, the number of TH-positive neurons in the substantia nigra was significantly reduced in the subacute PD mouse model established in this study, as shown in Fig. 1b and Fig. S1b, c.

Idebenone, as a coenzyme Q10 analog, has antioxidant and free radical scavenging effects. The MPTP subacute PD mice treated with idebenone had significantly fewer TH-positive neurons lost by the MPTP injury, as shown in Fig. 1b, c. To detect the expression of TH more accurately, we detected the protein level of TH in the substantia nigra. Figure 1d shows that the TH level of the MSG group was significantly lower than that of the control group. After treatment with idebenone for 21 days, the TH protein level of ITG was lower than that of the control and higher than that of MSG. Figure 1e is a quantitative analysis of the protein level, expressed as the ratio of the TH protein expression level to the β-actin protein expression level.

### Idebenone improves motor dysfunction and learning and memory ability in an MPTP-lesioned mouse model

MPTP can cause oxidative stress damage to mitochondria substantia nigra dopaminergic neurons, which leads to movement, learning, and memory disorders. Idebenone has the function of protecting mitochondria. We used the rotarod test and water maze test to study the effect of idebenone on the movement, learning, and memory impairment of the PD model.

**Fig. 2:**
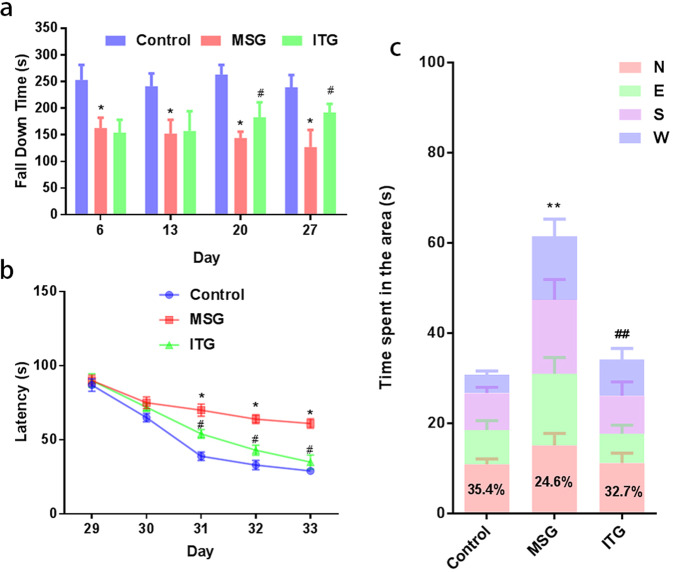
Effects of idebenone on motor and learning and memory impairment. **a** Rotarod test. Effect of idebenone treatment on motor dysfunction in PD model mice. **b** Water maze test. Effect of idebenone treatment on learning and memory ability of PD model mice (second phase). **c** Water maze test. Effect of idebenone treatment on learning and memory ability of PD model mice (third phase). *P < 0.05, **P < 0.01 vs. control, ^#^*P* < 0.05, ^##^*P* < 0.01 vs. MSG, *N* = 5.

We first used the rotarod test to analyze the movement disorder of the PD model, and the drop time was positively correlated with the coordination balance of the limbs and the grasping ability of the animals, as shown in Fig. 2a. The fall time of the control did not change significantly among the four experiments. In the first and second roller tests, the fall time of MSG and ITG groups were both shorter than that of the control. However, the difference between the two groups was not statistically significant. Interestingly, with the increase in the treatment time of idebenone, the fall time of the ITG group in the third and fourth roller tests was significantly longer than that of the MSG group.

A water maze was used to detect the effect of conditioned learning and memory impairment. The mice were subjected to the first stage of conditioned learning training on the 28th day, the second stage of detection on the 29th–33rd days as shown in Fig. 2b, and the third stage of detection on the 34th day as shown in Fig. 2c. The results showed that there were no significant differences between the three groups during the second stage on the 28th and 29th days. On the 30th day, the search time of the control was significantly faster than that of the MSG, and that of the ITG was also faster than the MSG. On the 31st day and the 32nd day, there was no significant difference between the control and ITG, and there was no significant decrease in the latency time among the MSG, control, and ITG. In the third stage, the histogram was divided into four regions. The N region was the region where the platform was previously located, and all of the time in the four regions was the time spent entering the water from the W region to reach the former platform region and staying there for 2 s. The total time for the control and ITG was not significantly different, but the MSG was different from the control and ITG. The ratio of residence time in the N region to the total time can reflect the memory ability of mice in searching for platform regions. The control and ITG accounted for 35.4% and 32.7% of the total time, respectively. Although the MSG stayed longer in the N region, it only accounted for 24.6% of the total time.

### Effect of idebenone on autophagy of substantia nigra neurons in a PD animal model

**Fig. 3:**
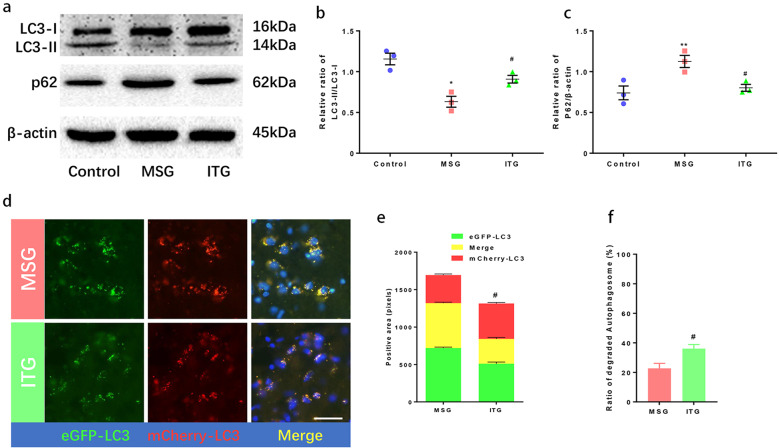
Effects of idebenone on autophagy in substantia nigra neurons. **a** Protein immunoblotting at the levels of LC3 and p62 in mouse midbrain substantia nigra. **b** Statistical analysis of LC3II/LC3I protein levels in the substantia nigra of mice. **c** Statistical analysis of protein quantity at p62 level in mouse midbrain substantia nigra. **d** LC3 immunofluorescence in substantia nigra of eGFP-mCherry-LC3 mice: green fluorescence eGFP-LC3, red fluorescence mCherry-LC3, yellow fluorescence merge. Bar = 100 μm. **e** Fluorescence intensity statistics of LC3 neurons in the substantia nigra of eGFP-mCherry-LC3 mice. **f** Ratio of autophagosomes that were successfully degraded *P < 0.05, **P < 0.01 vs. control, ^#^*P* < 0.01 vs. MSG, *N* = 3.

MPTP often leads to neuronal oxidative stress damage and mitophagy impairment. However, there are few studies on the effect of idebenone on MPP+-mediated autophagy. To study the effect of idebenone on autophagy, we detected the levels of LC3-II/LC3-I and p62 in MSG and ITG by western blotting, as shown in Fig. 3a. Compared with the control, the ratio of LC3-II/LC3-I in the MSG group decreased significantly, as shown in Fig. 3b. Although ITG decreased compared with the control, the increase in the ratio was also significant compared with MSG. p62, as the receptor of the vesicles to be degraded by autophagy, is used to detect the autophagy level. When autophagy is impaired, p62 will have an upward trend. MPTP treatment could significantly increase p62. ITG treatment with idebenone reduced the expression level of p62, and there was no significant difference compared with the control (Fig. 3c).

Green fluorescent tag is intolerant to acid, and eGFP labeled LC3 binds to lysosomes to reduce the level of green fluorescence, while mCherry labeled red fluorescence is not affected by lysosome acidity. Therefore, LC3 co-labeled with eGFP-mCherry can express the ratio of autophagosomes that are successfully degraded by fusion with lysosomes to all autophagosomes. The red fluorescence level minus the yellow fluorescence level/red fluorescence level was used to detect autophagic flux. We examined the autophagic flux of midbrain neurons in eGFP-mCherry-LC3 mice. Figure 3d shows the distribution of eGFP-labeled and mCherry-labeled LC3 in midbrain neurons. The mCherry red fluorescence represents all autophagosomes, and yellow fluorescence is the merged result of red fluorescence mCherry and green fluorescence eGFP. Figure 3e shows the statistical values of the positive pixels of various colors, and Fig. 3f shows the ratio of autophagolysosomes that were successfully degraded. ITG treated with idebenone showed a significant increase compared with MSG.

### Idebenone affects the detection of Parkin and PINK1 levels of Parkinson-related proteins

**Fig. 4:**
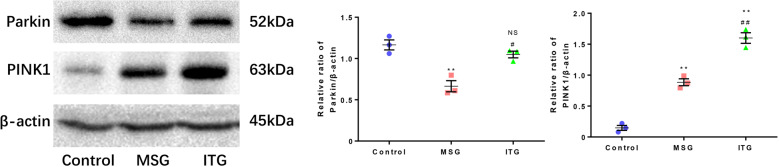
Idebenone activates the mitochondrial autophagy Parkin/PINK1 pathway. **a** Protein immunoblotting of the Parkin and PINK1 levels in the mouse midbrain substantia nigra. **b** Statistics of Parkin protein levels in the substantia nigra of the mice. **c** Statistical analysis of the PINK1 protein level in the mouse midbrain substantia nigra **P < 0.01, NS not significant vs. control, ^#^*P* < 0.05, ^##^*P* < 0.01 vs. MSG, *N* = 3.

Parkin and PINK1 are closely related to hereditary Parkinson’s disease and are very important for mitochondrial autophagy. Idebenone has a positive effect on mitochondrial stress, while MPTP causes mitochondrial oxidative stress damage. Therefore, we investigated the effects of idebenone on the PD-related genes Parkin and PINK1 in the PD model. We used MPTP subacute PD model mice to analyze the protein levels of Parkin and PINK1 in the substantia nigra of the midbrain (Fig. 4a). The results showed that the expression level of Parkin in the MSG group was significantly lower than that in the control group, and the expression level of Parkin in the ITG group was higher than that in the MSG group (Fig. 4b). The expression level of PINK1 in the MSG group was significantly higher than that in the control group. Interestingly, the ITG group had the highest levels of PINK1 relative to the control and MSG groups (Fig. 4c).

### Idebenone affects the mitophagy regulatory factors VDAC1 and BNIP3

Idebenone can affect the expression of Parkin and PINK1. Related studies have shown that VDAC1 and BNIP3 are closely related to Parkin/PIKN1-mediated mitophagy. We studied the pathway by which idebenone regulated mitophagy in the PD model by analyzing the protein levels of VDAC1 and BNIP3.

**Fig. 5:**
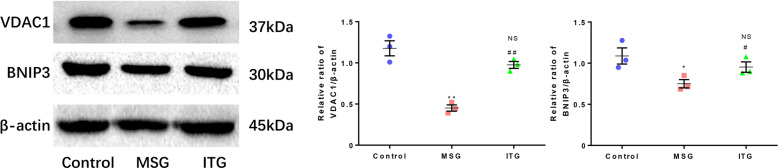
Effect of idebenone on mitochondrial membrane proteins VDAC1 and BNIP3. **a** Protein immunoblotting of the VDAC1 and BNIP3 levels in the mouse midbrain substantia nigra. **b** Statistics of VDAC1 protein levels in the substantia nigra of mice. **c** Statistical analysis of the BNIP3 protein level in the mouse midbrain substantia nigra *P < 0.05, **P < 0.01, NS not significant vs. control, ^#^*P* < 0.05, ^##^*P* < 0.01 vs. MSG, *N* = 3.

The expression levels of VDAC1 and BNIP3 in the substantia nigra of the MPTP subacute PD model mice are shown in Fig. 5a. The expression level of VDAC1 decreased in the MSG group compared with that in the control group. The expression level of VDAC1 in the ITG was not significantly changed compared with that in the control group but it was higher than that in the MSG group (Fig. 5b).

The expression of BNIP3 did not change significantly, but it was lower in the MSG group than in the control. In the ITG group treated with idebenone there was a significant increase of BNIP3 compared with MSG (Fig. 5c).

**Fig. 6:**
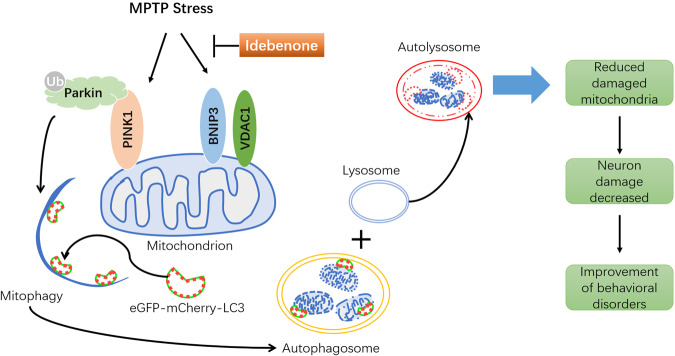
The effect of idebenone on mitophagy in MPTP mice. Idebenone affects Parkin/PINK1-mediated mitophagy by regulating BNIP3 and VDAC1 in MPTP-induced subacute PD eGFP-mCherry-LC3 mice.

Figure 6 shows that idebenone can affect Parkin/PINK1-mediated mitochondrial autophagy through VDAC1 and BNIP3, protect the mitochondria, restore the autophagic flux of damaged mitochondria to normal, reduce the number of damaged mitochondria and improve their behavioral disorders.

## Discussion

The MPTP mouse model is a commonly used PD model induced with chemical damage, and the pathological mechanism is that the number of dopaminergic neurons in the substantia nigra of the mice decreases. The model is divided into acute and subacute phases. The modeling cycle of the acute model is shorter, and the number of substantia nigra dopaminergic neurons decreases sharply [16]. However, the mortality rate of the mice is higher, their behavior changes rapidly, and the duration is shorter during the modeling injection process, which has a greater impact on the protective effect study of drugs [17]. Therefore, we chose a milder subacute model in which the number of TH-positive neurons in the subacute model reached a stable state after 21 days.

Rotarod tests can intuitively show the balance, grip, coordination, and movement ability of mice [18]. The fall time is inversely proportional to the severity of their movement disorder [19]. Our results showed that the movement disorder of the MSG group was significantly worse than that of the control group, which is consistent with previous behavioral studies on the MPTP mouse model. The daily intragastric administration of idebenone showed a trend of recovery from dyskinesia on the 20th day, but it was not statistically significantly different from MSG. There was no significant difference in dyskinesia about daily intragastric administration of idebenone showed between the mice without MPTP injury and the control group in Fig. S1d. Interestingly, it was statistically significant on the 27th day, but ITG itself had no significant difference between the 20th and 27th days. The reason for this phenomenon may be that the dyskinesia of MSG continuously worsened and reached its lowest level on day 27. The protective effect of idebenone on mitochondria can protect some TH-positive neurons from death. Although the scope of improvement is limited, it can appear to be significant in the later stages of motor disorder detection.

The water maze test is a common experiment to detect the ability of conditional learning and memory [20]. During the training, the mice can learn how to find the platform in the least amount of time. The learning and memory abilities of mice were measured by detecting their time spent lingering in the area where the platform used to be. The shorter the time spent in the water maze, the stronger their learning and memory abilities [21]. The 28th day of training and learning was set in the first stage (data not shown). The results of the second phase, 29th–33rd days, showed that the control group had a significantly reduced time spent in the pool within three days that reached a stable lower value on the 31st–33rd days. The trend of the MSG time reduction was very slow, with a significant decrease on the 30th day and no significant change on the 31st–33rd days. The ITG curves after treatment with idebenone were smooth and decreased without dramatic changes.

The third phase of the water maze experiment can intuitively reflect the influence of the different treatments on learning and memory ability. The platform was set in the N area. After the platform was removed in the third phase, the time proportion of mice wandering in the N area to find the removed platform can reflect the memory ability of the mice. Staying for 2 s in the area where the platform used to be terminated the test. The time spent was inversely proportional to their memory. Our results showed that idebenone improved learning and memory in PD mice. However, the comprehensive factors of learning and memory ability are complex and closely related to the degree of education. Therefore, these conclusions need to be further tested during the course of PD.

MPTP stress can cause changes in mouse brain neuron autophagy [22]. Our results confirmed the previous conclusions. Compared with that of the control group, the LC3II/LC3I ratio of the MPTP group was significantly decreased, and this decrease was reversed by idebenone. p62 is a substrate of autophagy, and low levels of p62 are often considered a sign of the smooth progression of autophagy. Compared with the control group, the MPTP group had higher p62 levels, and p62 also decreased after idebenone treatment. To further study the effect of idebenone on autophagy, we used LC3 dual fluorescence-labeled mice to verify the level of autophagic flux. Our results showed that idebenone could increase the degradation ratio of autophagolysosomes, so we believe that idebenone can change the autophagy level of substantia nigra neurons by promoting autophagic flux.

Due to the mitochondrial protective effect of idebenone itself [23] and the role of Parkin/PINK1 in mitophagy in PD [24], we were interested in whether the mitochondrial protective effect of idebenone is related to Parkin and PINK1. Parkin can participate in the expression of mitochondrial functional proteins (subunit complexes I and IV) [25]. When cell stress causes irreversible mitochondrial damage, Parkin promotes the clearance of damaged mitochondria through selective autophagy (mitophagy), and oxidative stress in the midbrain side of parkin knockout mice increases [26]. Overexpression of the PINK1 protein can protect against cell death induced by various toxins. When PINK1 is depleted, the susceptibility to cell death caused by stress will increase [27]. During stress, cells prevent mitochondrial dysfunction by acting downstream of PINK1, coordinating mitochondrial quality control mechanisms, and removing dysfunctional mitochondrial components [28]. Depending on the severity of mitochondrial dysfunction, the activity ranges from preventing apoptosis and stimulating mitochondrial biosynthesis to regulating the mitochondrial dynamics, and eliminating severely damaged mitochondria through mitochondrial phagocytosis.

MPTP stress leads to the degradation of a large number of damaged mitochondria in the cell, and thus the autophagic flow is blocked [29]. The damaged mitochondria cannot be degraded smoothly, leading to more damaged mitochondria. BNIP3 and LC3 can interact and selectively remove damaged endoplasmic reticulum and mitochondria through autophagy [30]. Our results indicate that the mitochondrial protective effect of idebenone in PD is related to the Parkin/PINK1 pathway and that idebenone can promote the activation of autophagic flux.

In conclusion, our results indicate that idebenone can improve motor dysfunction, learning, and memory in Parkinson’s model mice by regulating mitophagy. Idebenone may be a promising drug for improving the mitochondrial function during Parkinson’s disease.

## Materials and methods

### Animals

C57BL/6J male mice, 6–8 weeks old, weighing 20–25 g, were maintained in alternating 12 h light and dark cycles at 25 °C with free access to food and water. For details of the eGFP-mCherry-LC3 mice, refer to our previous study [31]. This study was approved by the Ethics Committee/Institutional Review Board of the First Affiliated Hospital of Henan University of Science and Technology. The guidelines of the NIH’s Guide for the Care and Use of Laboratory Animals and the International Association for the Study of Pain (IASP) were followed. According to the previously published protocols for the establishment of PD animal models [16], a subacute MPTP mouse model (intraperitoneal injection of 30 mg/kg MPTP once a day for five consecutive days) was adopted. This model is currently widely used. According to previous studies, we used a 200 mg/kg dose [32] to give the mice a single daily gavage of idebenone dissolved in water (20 mg/ml idebenone in water) for 21 days after the successful establishment of the subacute MPTP model.

The experiment was divided into three groups: 1) The control group was intraperitoneally injected with saline once a day for 7 days. Idebenone was gavaged once a day for 21 days. 2) The MPTP subacute model group (MSG) was intraperitoneally injected with MPTP once a day for 7 days. This group received daily gavage of the vehicle (water) for 21 days. 3) The idebenone treatment group (ITG) was intraperitoneally injected with MPTP once a day for 7 days. Idebenone was given by gavage once a day for 21 days [33].

### Rotarod test

After 2 h of idebenone treatment, the mice were placed on a 7 cm diameter rotating rod, and an accelerated mode test was performed from 4 to 40 rpm for 5 min. The average latency time (i.e., the first fall from the rod) was recorded. The roller experiment was carried out on the 6th, 13th, 20th, and 27th days (Fig. 1, blue dotted box). Each mouse was tested three times with an interval of 30 min and the average was taken. The experiments were all performed in the same quiet and well-lit laboratory environment.

### Water maze test

The water maze (Shanghai Xinran) is a black tank container 120 cm in diameter, 50 cm in height, and 25 cm in depth. Milk was added to the water to enhance the contrast between the C57 mice and the background. The water was heated by a thermostat and maintained at approximately 22 °C. Four differently shaped markers were evenly distributed along the edge of the pool wall, dividing the pool into four virtual quadrants (regions N, E, S, and W). The platform was placed approximately 1 cm under the water and fixed in the N region. The mice were put into the water at the edge of the pool on the opposite side from the platform (W region). Then, the time it took the animals to find the underwater platform (latency) was recorded.

In the first phase (training phase, 28th day), each mouse was gently placed on the water maze platform facing the wall and the mouse was guided to observe the shape marker for 90 s. Then, the mouse was placed in the water from the W region. If the platform was not found during the training, the mouse was guided to the platform and maintained there for 20 s for observation and learning. In the second phase on the 5th day (29th day to 33rd day), each mouse was gently faced toward the tank wall of the W region of the water maze, and the mouse swam and looked for the platform. If the mouse successfully found the platform and stayed on the platform for 2 s, the experiment was stopped and the time was recorded. If the mouse did not find the platform within 90 s, it was recorded as 90 s and the experiment was ended. In the third phase (platform crossing phase, 34th day), the platform was removed, and each mouse was gently placed in the water maze in the W region. The number of times the mouse crossed the area, where the platform used to be within 90 s while staying in the platform area for 2 s was recorded. The learning ability and the spatial memory ability of the mice were evaluated by the platform search latency and crossing-platform times. The data were recorded and analyzed using Anymaze software (Stoelting).

### Immunoblotting

Mice in each group were sacrificed to collect substantia nigra brain tissue. It was placed in RIPA buffer containing PMSF, ground on ice and extracted for 20 min. The supernatant containing the protein was obtained by centrifugation at 4 °C at 12,000 × *g* for 15 min. The protein concentration was determined by a BCA Protein Assay Kit (Cwbio, CW0014S). The total protein extract was mixed with loading buffer and the mixture was separated on a 12% glycine SDS-PAGE gel and transferred to a 0.22 μm PVDF membrane. The membranes were blocked with 5% PBST skim milk solution at 37 °C for 1 h. Then, the membranes in PBST with 0.5% skimmed milk were incubated overnight at 4 °C with primary antibodies against LC3B (Abcam, ab192890), P62 (ProteinTech, 18420-1-AP), PINK1 (Santa Cruz, sc-518052), Parkin (Santa Cruz, sc-32282), BNIP3 (Abcam, ab109362), beta-actin (Cwbio, CW0096M), and VDAC1 (ProteinTech, 66345-1-LG) at 1:1000 dilution. The PVDF membrane was then washed with PBST three times, 5 min each time, and incubated with a 1:5000 dilution of the secondary antibody (Cwbio, CW0102S, CW0103S) at room temperature for 1 h. The membranes were then washed with PBST three times for 5 min each time. All experiments were repeated at least three times to ensure accuracy. The PVDF membrane was developed by ECL and a Chemi-Doc-XRS system (Bio-Rad). Determination of the fluorescence intensity of the protein bands was performed by ImageJ software.

### Immunofluorescence

Mice were perfused with 4% paraformaldehyde at 4 °C after sacrifice. After complete brain dissection, gradient sucrose solution was used for dehydration. The mouse brain was embedded in O.C.T. compound (Sakura, 4583) and coronally sliced at −25 °C (20 μm) in a freezing microtome. The slices were washed with PBS, and slices of the substantia nigra were selected by soft brushing. The slides were blocked with goat serum (Solarbio, SL038) for 30 min, then placed in 1% BSA and anti-TH antibody (ProteinTech, 25859-1-AP) at 1:500 and incubated overnight at 4 °C. The fluorescent secondary antibody anti-488 (Bioss, bs-0295G-AF488) was diluted at 1:1000 in 1% BSA and incubated with the slides at room temperature for 1 h (eGFP-mCherry-LC3 mice were stained with DAPI for 5 min after brain sectioning). After washing with PBS three times, the slides were sealed with glycerin. All images were obtained by fluorescence microscopy (Olympus, BX53).

### TH positive neuron count

According to the stereological analysis method published by Katrine Fabricius [34], we selected sections from bregma −2.46 mm to −4.04 mm, and used frozen sections with a thickness of 20 µm (ssf, every 30th section) and a frame area of S (15,000 μm^2^), The frames were placed at equidistant X, Y-steps (264 μm) within the region of interest on each section. The total number of TH-positive neurons in each slice was counted and recorded as T; then, the total number of neurons was expressed as ${{{{N}}}} = (\frac{1}{{{{{\mathrm{ssf}}}}}} \ast \frac{{264}}{{{{\mathrm{s}}}}} \ast {{{{T}}}})/2$.

### Statistical analysis

GraphPad Prism 7.0 was used to analyze the data. All experiments were repeated three times independently, and the data conformed to a normal distribution, which was expressed as the mean ± standard error (SEM). An unpaired *t*-test was used to compare two groups, one-way or two-way ANOVA was used to compare multiple groups, and Tukey’s post hoc test was applied. A difference of *P* < 0.05 was considered statistically significant.

## Supplementary information

supplementary legends

supplementary Figure 1

supplementary Figure 2

## Data Availability

The original contributions presented in the study are included in the article material, further inquiries can be directed to the corresponding authors.
