# Supplementary material for: Idebenone improves motor dysfunction, learning and memory by regulating mitophagy in MPTP-treated mice
Source: Cell Death Discov. 2022 Jan 17;8:28. doi: 10.1038/s41420-022-00826-8 (PMC8764058; doi:10.1038/s41420-022-00826-8)
Supplement: Supplementary file 1 — supplementary legends [file 41420_2022_826_MOESM1_ESM.docx]

**Supplementary legends**

Supplementary Figure 1

Effect of idebenone on dopaminergic neurons in the substantia nigra of C57BL/6j model.

**a**: Mouse model, idebenone treatment and behavioral processing diagram. **b**: Immunofluorescence of dopaminergic neurons in the substantia nigra of the mice. Bar=200 μm **c**: Statistics of dopaminergic neurons in the substantia nigra of the mice. **d**: Rotarod test. Effect of idebenone treatment on motor dysfunction in mice.

Supplementary Figure 2

Effects of idebenone on TH and mitophagy related protein level in substantia nigra neurons.

**a**: Protein immunoblotting at the levels of TH, LC3, p62, Parkin, PINK1, VDAC1 and BNIP3 in mouse midbrain substantia nigra. **b**: Statistical analysis of TH, LC3, p62, Parkin, PINK1, VDAC1 and BNIP3 protein levels in the substantia nigra of mice.
