# Supplementary figures and images for: Idebenone improves motor dysfunction, learning and memory by regulating mitophagy in MPTP-treated mice
Source: Cell Death Discov. 2022 Jan 17;8:28. doi: 10.1038/s41420-022-00826-8 (PMC8764058; doi:10.1038/s41420-022-00826-8)

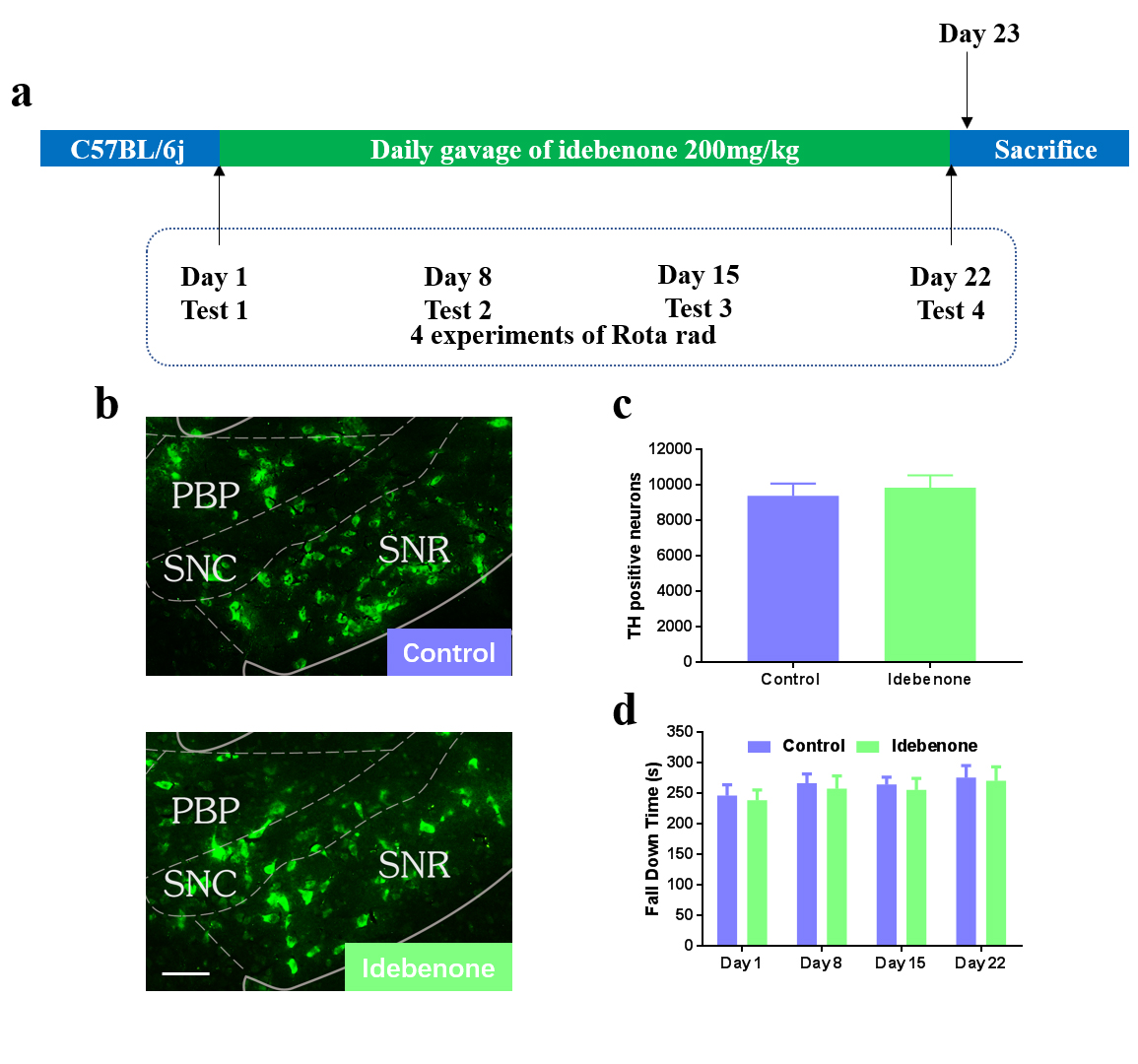

Supplement: Supplementary file 2 — supplementary Figure 1 [file 41420_2022_826_MOESM2_ESM.jpg]

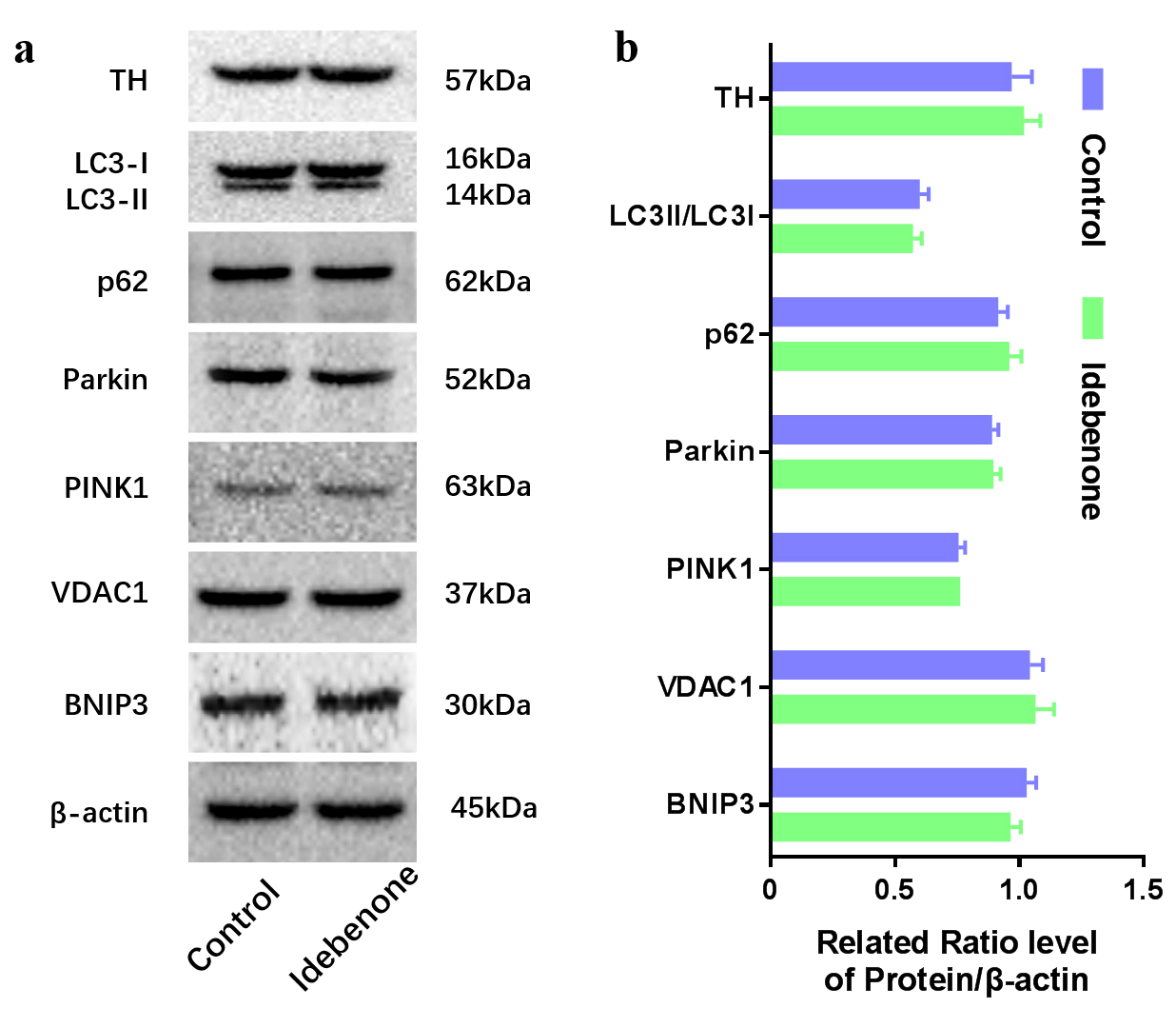

Supplement: Supplementary file 3 — supplementary Figure 2 [file 41420_2022_826_MOESM3_ESM.jpg]
